# Mechanisms of resistance to anti-angiogenic treatments

**DOI:** 10.20517/cdr.2019.39

**Published:** 2019-09-19

**Authors:** Francesco Pezzella

**Affiliations:** Nuffield Division of Clinical Laboratory Science, Radcliffe Department of Medicine, University of Oxford, John Radcliffe Hospital, Oxford, OX3 9DU, UK.

**Keywords:** Angiogenic tumours, non-angiogenic tumours, anti-angiogenic treatment, resistance, hypoxia, vascular co-option

## Abstract

Hailed as the cancer treatment to end all the resistance to treatment, anti-angiogenic therapy turned out to be not quite what was promised. The hope that this therapeutic approach would not have suffered by the phenomenon of resistance was based on the fact that was targeting normal vessels rather than tumour cells prone to mutation and subject to drug induced selection. However, reality turned out to be more complex and since 1997, several mechanisms of resistance have been described to the point that the study of resistance to these drugs is now a very large field. Far from being exhaustive, this paper presents the main mechanisms discovered trough some examples.

## Introduction

Solid tumours need a blood supply and a large body of evidence has previously suggested that they can only grow behind a few millimetres in diameter if they induce the development of new blood vessels, a process known as angiogenesis. Based on this hypothesis, it was proposed that anti-angiogenic drugs should be able to suppress the growth of all solid tumours by killing off the blood supply^[1]^. However, after many clinical trials with anti-angiogenic agents, we now know that this is not always the case. Unfortunately, hope for a “pan-cancer” anti-angiogenic therapy^[2]^ had been greatly diminished with the finding that the efficacy of vascular endothelial growth factor (*VEGF*) signaling pathway inhibitors is modest and is restricted to certain advanced-stage cancers only. Crucially, overshadowed until now, there is an increasing body of research, spanning now 25 years, demonstrating that not all the tumours are angiogenic. There are also non-angiogenic tumours that grow by exploiting pre-existing blood vessels of the surrounding nonmalignant tissue^[3]^ using a process called vessel co-option^[4]^. This is a frequently overlooked mechanism of tumour vascularization that can mediate disease progression and metastasis. Its identification led also, for the first time, to raise the hypothesis that resistance to anti-angiogenic treatments can occur^[5]^. Until than this type of cancer treatment was regarded as unable to meet or induce any type resistance^[6]^.

## Clinical findings

In 1971, Folkman^[1]^ proposed that new blood vessel formation (angiogenesis) is necessary for the progression of solid tumours beyond a size of a few millimetres cube and that blocking angiogenesis in tumours might be an effective means of maintaining or inducing dormancy and preventing metastasis. The premise of this hypothesis was that cancers cells chances of survival are dependent on their ability to induce formation of new vessels to deliver oxygen as all the pre-existing vessels were assumed to be destroyed by the neoplastic process. In the following years, many studies seemed to support this hypothesis. The direct association between the microvascular density observed in the neoplastic tissue and tumour aggressiveness^[7,8]^, the identification of the angiogenic factors of the *VEGF* family^[9,10]^ and the discovery that they are widely upregulated in human tumours^[11-14]^ seemed to provide more evidences. The report in the mid 90s that angiostatin could induces astonishing levels of tumour regression in mouse models^[15,16]^ further pushed the idea that antiangiogenic agents could treat patients with both early and advanced malignancies. Based in large part on these data, induction of angiogenesis has been considered as a hallmark of cancer since 2000^[17,18]^. It also emerged at the time the idea that, as the targeted vessels were not neoplastic, this type of cancer treatment could be immune from resistance^[6]^.

In spite of all the high expectations, clinical trials produced disappointing results. For example, in high grade glioma, a Cochrane Review concluded that anti-angiogenic drugs do not improve significantly overall survival and there are no evidences to support this therapeutic approach^[19]^. However, in these patients, bevacizumab treatment can relieve symptoms by reducing the severity of intracranial oedema^[20]^. In advanced breast cancer improvement in disease free but crucially not overall survival has been seen while the results in early breast tumours are inconclusive^[21]^. Anti-angiogenic treatment has been instead effective in improving the outcome of patients with metastatic^[22]^ colorectal cancer, although the improvements achieved are in the range of months rather than years. No benefit has been instead found for patients with early colorectal cancer^[23]^. Disappointing the results in Small Cell Lung cancer^[24]^ while positive results have been reported in non-Small Cell Lung cancer, although the improvements in progression free and overall survival observed are, again, modest^[25]^.

## Mechanisms of resistance

The first evidence that cancer could be resistant to anti-angiogenic treatment was published in 1997 when non-angiogenic tumours were recognised and formally described for the first time in the lung^[5]^. In the following years, a number of mechanisms of resistance have been discovered. Resistance to antiangiogenic therapies can be intrinsic, when it is observed at the beginning of the treatment, or acquired, i.e., that it affects the relapsing disease after an initial response to therapy^[26,27]^. Here are illustrated the main mechanisms.

## Non angiogenic growth by vascular co-option

**Figure 1:**
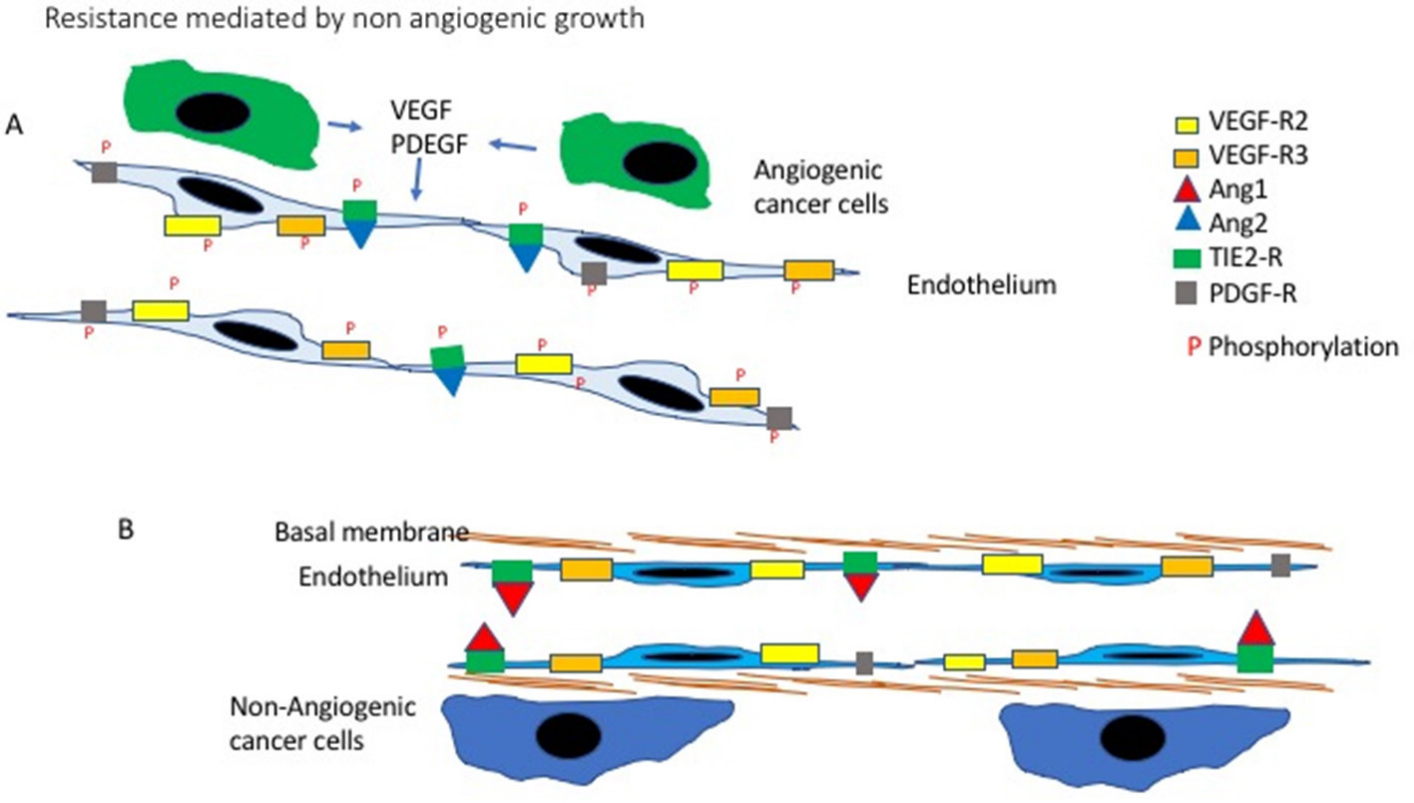
Non-angiogenic tumours: rational for resistance to anti-angiogenic treatment. A: Newly formed vessel. When cancer cells induce angiogenesis, new vessels sprout from the pre-existing one. These new vessels are lined up by newly formed proliferating endothelial cells. Under the influence of the angiogenic factors released by the cancer cells, like *VEGF* and PDEGF, the endothelial cells express higher levels of the relevant receptors. Following binding with their ligands, the intracellular portion of the receptors is phosphorylated activating the downstream intracellular pathways. A third angiogenic mechanisms is also present in this example: Angiopoietin 2 compete with Angiopoietin 1 and binds with the Tie receptor. Angiopoietin 1 maintains the endothelial cell quiescent, by taking its places Angiopoietin 2 trigger endothelial proliferation. In this situation compounds blocking *VEGF* or inhibitors of the Tyrosine kinases, blocking the activation of receptors like *VEGFR2*, can be effective; B: A co-opted pre-existing normal vessel. In non-angiogenic tumours the cells do not trigger angiogenesis but grow by exploiting the pre-existing vessels. These are lined by quiescent mature endothelial cells those few receptors for angiogenic factors remain inactive as no angiogenic stimuli are present. The Tie2 receptors remain linked to Angiopoietin 1 which maintain the vessel quiescent. Much is still to be learned about the biology of this system but is it is possible to appreciate how antibodies blocking *VEGF* or tyrosine kinases inhibiting angiogenesis, will not have any effects as the pathways they target are not contributing to the growth of these tumours. *VEGF*: vascular endothelial growth factor; PDEGF: platelets derived endothelial growth factor

Non-angiogenic cancers are early or advanced symptomatic tumours which grow by exploiting pre-existing vessels by means of vascular co-option. These neoplasms can be very aggressive but as they are lacking angiogenesis, it was immediately evident that anti-angiogenic treatment could have been completely ineffective in these patients and, for the first time, was suggested that this type of therapeutic approach could face resistance, as all the others do^[5]^. That non angiogenic growth, by vascular co-option is a mechanism of resistance to anti agiogenic treatmentrs has been now proved by a large numebr of clinical and pre-clinical studies. The rational behind this mechanism is illustrated in Figure 1.

Comparative clinco pathological investigations have confirmed that intrinsec and extrinsec resistance can be modulated by non angiogenic growth. The secondary location of breast carcinomas to the lung^[28-30]^ liver^[31,32]^, lymph nodes^[33]^, skin^[34]^ and brain^[35-37]^ is frequently due to non-angiogenic growth. This conclusion can explain why phase III clinical trials in patients with advanced breast cancer involving sunitinib or bevacizumab as anti-angiogenic agent, either alone or with chemotherapy, have not been as successful as expected.

Similar observations have been provided by the study of brain tumours. Post mortem studies in patients treated with Cediranib, an inhibitor of *VEGF* receptor 2 (*VEGFR2*) tyrosine kinases, or Bevacizumab regimen^[38,39]^ showed that the glioma cells were growing around pre-existing vessels. Contrast-enhanced MRI found, having administered bevacizumab, that spreading gliomas had a non-enhancing pastern consistent with invasive perivascular malignant progression^[40,20]^. Again, patients with metastatic colorectal carcinoma to the liver have a higher rate of response to bevacizumab plus chemotherapy when they have angiogenic metastases, while the poor responders have mostly non angiogenic secondaries^[30]^. The responders have also a significantly improved OS compared to the poor responder with non-angiogenic lesions (HR 3.50, 95% CI 1.49-8.20; *P* = 0.0022) indicating an association between vessel co-option and a poor response to antiangiogenic therapy.

**Figure 2:**
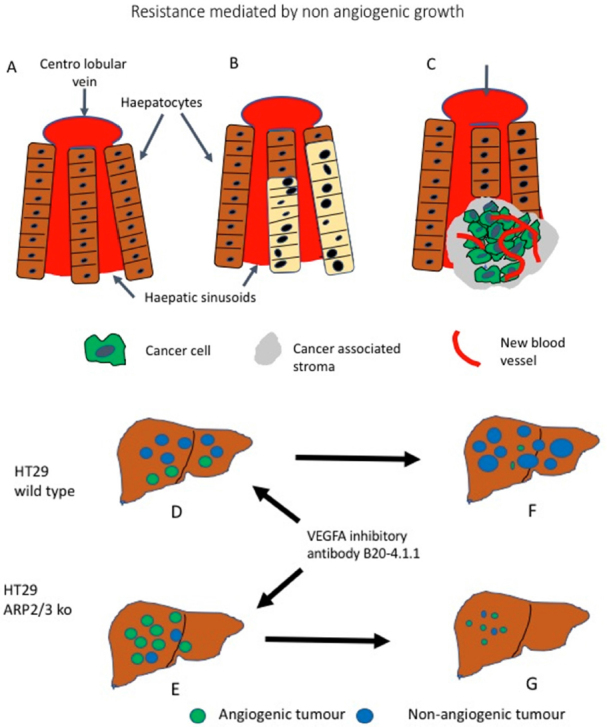
Non-angiogenic tumours are a cause for resistance to anti angiogenic treatment. A: Schematic representation of normal liver; B: a non-angiogenic liver metastases in which the liver architecture is preserved, the metastatic cells (beige) take the place of the hepatocytes exploiting the liver sinusoidal vascular system; C: an angiogenic metastases (green) grows by destroying the liver tissue, new vessels (in red) are sprouting providing the blood flow; D: HT29 Wild Type colorectal cancer cells produces metastases when injected into the mouse liver and the majority of them are non-angiogenic (blue). If HT29 cells with silenced ARP2/3 complex are injected; E: the metastatic growth will be predominantly angiogenic (green). When treatment with an anti *VEGF* antibody is performed, the angiogenic metastases of wild type tumours regresses, but the non-angiogenic lesions progresses; F: However, in tumours made up by HT29 cells with silenced ARP2/3 complexes the angiogenic metastases respond; G: Because of the silencing of the ARP2/3 complexes, the non-angiogenic metastases do not develop further. (Based on^[32]^)

Frentzas *et al*.^[32]^ have provided evidences in a mouse model that co-option is effectively the cause for the resistance described in the above clinical examples. As vascular co-option requires for the cancer cells to be motile, the authors scrutinised the role of ARP2/3 complex. The actin-related protein 2 (ARP2; encoded by ACTR2) and ARP3 complex (known as the ARP2/3 complex) is involved with the nucleation-promoting factor into the nucleation of Actin filaments, leading to cell motility. In cancer, the ARP2/3 complex is well known as one of the key factors promoting invasion and metastases^[41]^. First Frentzas *et al*.^[32]^ analysed the expression of the ARPC3 component of the complex by immunohistochemistry in the liver metastases proving higher expression in the non-angiogenic lesions compared to the angiogenic once. Than they moved to investigate the role of ARP2/3 in co-option using a mouse model of metastases in which the human colorectal cancer cell line HT29 is injected into the liver [Figure 2]. Metastases are than produced with both angiogenic and non-angiogenic areas. The authors than successfully knocked down ARP2/3 in this cell line using two different short hairpin mRNA. Depleted of ARP2/3 the cell motility was impaired but not its proliferation. Once injected into the liver these cells still produced metastatic lesions but prevalently angiogenic.

The mice with HT29 wild type metastatic disease, mostly non angiogenic, did not responded well to treatment with the *VEGFA* inhibitory antibody B20-4.1.1, however this antibody was effective in significantly reducing the neoplastic bulk in mice injected with ARP2/3 negative HT29 cells. This indicate that abrogation of motility and subsequent co-option, drive the cells to use angiogenesis and the lesion is therefore sensible to anti angiogenic compounds. It can be concluded that is the non-angiogenic nature of the tumour causing resistance and this can be reversed by preventing co-option happening^[32]^. Association between non-angiogenic tumours and resistance has been also illustrated in a different mouse model in which an orthotopic model of hepatocellular carcinoma is initially sensitive to Sorafenib, another Tyrosine Kinase inhibitor blocking the *VEGF* induced signalling, only to develop resistance after one month, by switching to an invasive phenotype, with upregulation of EMT-associated genes and co-option of sinusoidal and portal-tract vessels^[42]^.

A first step in overcoming these problems would be an assessment of anti-angiogenic drugs in clinical trials where patients are selected according to predictive biomarkers (e.g., the vascular pattern). So far there have been no randomized phase III clinical trial of an antiangiogenic drug guided by biomarkers reflecting the type of vascularization present (e.g., newly formed vessels versus vascular co-option)^[1,43]^. This stands in marked contrast to what has happened in different situations in which clinical trials were designed according to predictive biomarkers such as, e.g., trastuzumab in HER2-positive breast cancer patients. A second approach under inquiry is the combination of treatment against angiogenesis and against vascular co-option. This follow the observations that vascular co-option is a mechanism of resistance^[32]^ but the angiogenic status of a tumour can change during progression in both ways^[3,29]^. Therefore it is emerging that, as an angiogenic tumour treated with anti-angiogenic drugs can “escape” by turning non-angiogenic, also a non-angiogenic tumour treated with anti-co-option drugs could “escape” acquiring an angiogenic phenotype. The combined approach has been also suggested by animal model studies. Seaman *et al*.^[44]^ described that *CD276*, a highly conserved cell-surface protein, is overexpressed in many different types of cancer both on cancer cells and endothelium. The most interesting finding was that *CD276* was expressed on both newly formed and pre-existing vessels inside the tumours but not in normal vessels outside the malignant lesion and not during physiological angiogenesis. In a mouse model, a drug conjugated with anti *CD276* antibody, eradicated both large established tumours and metastases and improved long-term overall survival likely because of the targeting of both angiogenic and non-angiogenic intra-tumour blood vessels^[44]^.

## Hypoxia mediated resistance

**Figure 3:**
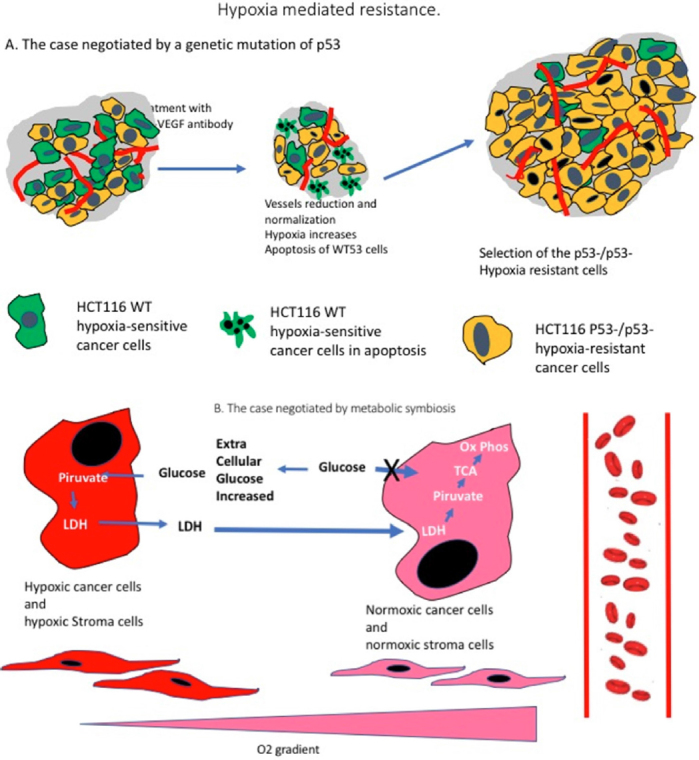
Resistance by Hypoxia. Following anti-angiogenic treatment, the decrease in number of vessels frequently leads to increased hypoxia levels. A: Mechanism driven by *p53* mutation. In the model investigated by Yu *et al*.^[47]^ the human colorectal cancer HCT116 cell line is used in its wild type and *p53*-/*p53*- versions. When a mouse carrying a xenograft produced by a mixture of HCTT16 WT and HCT116 *p53*-/*p53*- is treated with an anti *VEGF* antibody an initial shrinkage of the tumour occurs as most of the WT-hypoxia sensitive cells undergo apoptosis. However, the *p53*-/*p53*- cells, hypoxia resistant, continuous to growth producing eventually an even larger mass. (based on Yu *et al*.^[47]^); B: Mechanism driven by Metabolic Symbiosis. As the number of hypoxic cells increases, following treatment-induced vascular disappearance, the hypoxic cells by releasing LDH allows the normoxic cells, which internalise it and turn it into pyruvate, to improve their Oxidative Phosphorylation, decrease their consume of glucose and therefore leave more glucose available. for the hypoxic cells which have therefore the possibility to produce more energy and survive. The same phenomenon occurs for the tumour-associated stroma. (Based on ^[51]^and ^[52]^)

**Figure 4:**
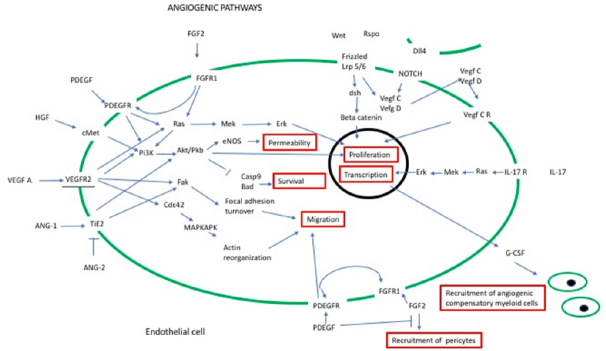
Redundant angiogenic pathways. Binding of *VEGFA* with is receptor *VEGFR2* lead to the activation of four main pathways. Two, the Ras/MAPK and the PI3K/AKT promotes proliferation of the endothelial cell, while the other two, Fak and Cdc42 pathways induces migration of the same cells. Proliferation and migration of the endothelial cells is necessary for angiogenesis to happen. However, angiogenic stimuli leading to these two events in the endothelium can also be independent from the *VEFG* action. As illustrated in this picture, PDEGF and *FGF2* also lead to activation of the Ras/MAPK pathways and therefore proliferation and on activation of migration. Angiopoietin 1 can activate motility as the Wnt Canonical pathway can trigger proliferation. (Based on data from^[81]^). HGF: hepatocyte growth factor; Mek: methyl ethyl ketone; eNOS: endothelial Nitric Oxide Synthase 3; MAPK: mitogen activated protein kinase; Erk: extracellular signal regulated kinase; PI3K/AKT: phosphoinositide 3-kinase/protein kinase B.

Some of the first, among other mechanisms of resistance discovered, have been those mediated by hypoxia^[45]^. It is a heterogeneous group, as different can be the causes of the better adaptation of a cell to hypoxia, but the common factor is that cells more equipped to survive in hypoxia, are more likely to remain vital and able to growth after treatment-induced reduction of the vascularity. In one of the first studies published, the cause is a genetic damage. Following the observation that *p53* negative neoplastic cells are more resistant to apoptosis induced by hypoxia^[46]^, Yu *et al*.^[47]^ investigated in a mouse model whether the increased hypoxia which follow the targeting of blood vessels, could select the growth of *p53* negative cancer cells [Figure 3A]. Mice were injected with human colorectal cancer cell lines HCT116 wild type, *p53* positive, and HCT116 *p53*-/*p53*-. Once the neoplasms had developed, the mice were treated with low dose vinblastine and the antibody DC101, against the anti-murine *VEGFR2*, or with the DC101 antibody alone. The HCT116 *p53*-/*53-* xenograft were slower to respond to both treatment schedules. In a second set of experiments xenografts were induced by injecting a mixture of an equal amount of HCT116 *p53*-/*53-* and Wild Type (*p53*+/*p53*+) cells. Following treatment with Vinblastim and DC101, a slowdown of the tumour growth compared to the negative control was found. However, these residual tumours were still growing and the percentage of *p53*-/*53-* cells in the tumour had increased as they demonstrated to have a selective advantage over the *p53*+/*p53*+ [Figure 4]. Finally, the authors showed that in an untreated xenograft induced by injecting 50%/50% mixture of *p53* positive and negative cells, the less hypoxic areas, closer to the vessels, were richer in *p53*+ positive cells than the sections of tumours more distant from the blood supply. This was due to a higher rate of apoptosis among the *p53*+/*p53*+ cells distant from the blood vessels. The author concluded that the genetic imprinting of the neoplastic cell can therefore be a cause of resistance to at least some types of antiangiogenic treatments^[47]^.

A second way in which hypoxia is believed to mediate resistance is after treatment with anti *VEGF* therapy. One example is that reported in a study by Paez-Ribes *et al*.^[48]^. In murine models of neuro endocrine pancreatic cancer and of glioblastoma, treatment with the anti-murine *VEGRF2* antibody DC101 is followed by reduction in volume of the tumours but, at the same time, the residual malignancy has a more invasive phenotype which persisted also after stopping treatment. In tumour cells with the *VEGF-A* gene deleted, a similar aggressive phenotype was found. Anatomical examination also revealed an increased number of metastatic lesions occurring. Similar results were found when, instead of the DC101 antibody, an anti-angiogenic tyrosine kinase inhibitor, Sunitinib, was employed to treat the tumours. As in the previous model, also in this one an increase in the number of hypoxic cells has been found suggesting again a link between post treatment development of hypoxia and escape from drugs targeting the new vessels^[48,49]^.

In a third study where treatment with Bevacizumab is followed again by hypoxia and increased invasiveness of glioma cells^[50]^ the authors dissect the mechanism by which hypoxia leads to invasiveness. In the neoplastic cell investigated, the higher levels of Hif1 which follows hypoxia induces, among others, the transcription of *ZEB2*. *ZEB2* protein overexpression leads to down regulation of *Ephrin B2* and, in this model the authors demonstrated that, following loss of *Ephrin B2* expression, the glioma cells become more invasive^[50]^.

A final example of how the increased levels of hypoxia caused by anti-angiogenic treatment can lead to resistance and tumour growth is the one which relay on metabolic symbiosis^[51]^. This is a process in which both cancer cells and tumour associated stromal cells located in the best oxygenated areas, collaborate with the more hypoxic cells in order to optimise their respective metabolisms and energy production [Figure 3B]. The more hypoxic the cell, the more glycolysis is used leading to production of pyruvate. As oxygen is scarce, most of the pyruvate is converted to lactate, instead of entering the tricarboxylic acid cycle (TAC). This excess of lactate is secreted in the extra cellular environment where it diffuses and is picked up by the more oxygenated cells which internalise the lactate in the cytoplasm, revert it to pyruvate and use it to further enhance the efficiency of their TAC and, consequently, of their oxidative phosphorylation. As the better oxygenate cells improve the use of their respiratory chain, their need for glucose decreases. As a consequence, more glucose is left available in extracellular compartment for the more hypoxic areas of the tumour^[51]^. Following anti-angiogenic treatment therefore, it has been observed that the establishment of this positive symbiotic loop allows the cells to remain viable and proliferate also in face of the dramatic increases of hypoxia which follow angiogenesis inhibition and collapse of part of the vasculature^[52]^.

How to overcome hypoxia mediate resistance has been the object of investigation for many years^[53]^ and its discussion would be too long for this review. Several the approaches currently under scrutiny. Some of the most interesting are those looking at targeting the associated metabolic changes^[54,55]^, targeting epigenetic changes, using drug associated nano particles^[56]^, use hypoxia imaging as predictive factor^[57]^ or use of small molecules inhibiting protein-protein interaction to target Hif1^[58]^.

## Redundancy of the angiogenic signals

The main angiogenic pathway is the *VEGF* one^[59]^ however it is not the only and, in its complexity, interacts with several other pathways. Recently in a very thorough review Gacche and Assaraf^[60]^ identified three main mechanisms of resistance due to redundancy of the angiogenic signals: 1) activation of pathways involving angiogenic factors other than *VEGF*; 2) replacement production of *VEGF* by non- neoplastic, stromal cells or 3) pericytes-driven angiogenesis. There are several angiogenic factors other than *VEGF* (for review by Gacche et al.^[60]^ and Ribatti^[49]^) and the main one, with their correspondent pathways, are illustrated in Figure 4. Among these pathways an important *VEGF*- independent angiogenic activity is provided by the interplay of fibroblastic growth factor (*FGF2*) and *PDGF-BB*^[61]^. *PDGF-BB* is a factor with strong chemotactic and mitogenic action on the pericytes while the *FGF2* induces proliferation mostly on the endothelium. In this study the authors demonstrate that the angiogenic factor *FGF2* has two actions on the endothelial cells: triggers proliferation, through the Ras/MAPK pathway and also induces higher levels of two PDEGF receptors: the alpha and the beta. In this way, the sensitivity of the endothelial cell to PDEGF is increased and, as consequence, also its motility is stepped up^[61]^. Instead while the presence of *FGF2* recruits and maintain pericytes, the additional expression of PDEGF inhibits their recruitment, making the vessels very leaky^[61]^. As this pathway is *VEGF* independent, it can maintain angiogenesis in presence of anti *VEGF* pathways drugs.

These factors providing redundant angiogenic signals have also other roles in cancer and many approaches to target them are being investigated. *FGF2* is widely involved in many types of cancer trough activation of the Ras/MAPK and PI3K pathways causing not only angiogenesis but also increased proliferation and metastatic spread^[62]^. Targeting *FGF2* is therefore one of the ways to overcome resistance to anti *VEGF* therapy^[63,64]^. PDGF also is involved in many aspects of the cancer cell biology and both pharmacological compounds and inhibitors of tyrosine kinases specific for this pathway are being investigated^[65,66]^. Finally, because of the emerging role of pericytes in maintaining intra tumour vessels viable, targeting these cells has become the latest approach investigated to overcome resistance to anti *VEGF* treatment^[67,68]^.

## Vascular heterogeneity

Endothelial cells sensitive to anti *VEGF* therapy rely on this pathway. However not all the endothelial cells are the same. In the human body differences exists according to the anatomical location and the type of vessels^[69,70]^. Inside cancer lesions vascular heterogeneity is even more pronounced, intra tumour vessels have a heterogeneous anatomical structure and endothelial phenotype^[71]^. Anatomically the main features causing heterogeneity are a variable degree of leakage and variable coverage by pericytes. The endothelial cells themselves are than heterogeneous as far as the genotype and the phenotype is concerned. Endothelial genotypic differences in mice models are due to the occurrence of aneuploidy and presence of abnormal centromeres, and the genetic defects, in this model, are not due to contamination form the tumour. Variable patterns of mRNA transcriptions have also been found which lead to a variable protein phenotype resulting in differences in behaviour and response to drugs and growth factors^[71]^. One case of heterogeneity is due the up regulation in endothelial cells, associated with tumours, of the PI3K/AKT pathway which has been reported in some intra tumour endothelial cells^[72]^, one of the consequences is the variable response to angiogenic factors like *VEGF* and to their blockage^[73]^.

**Figure 5:**
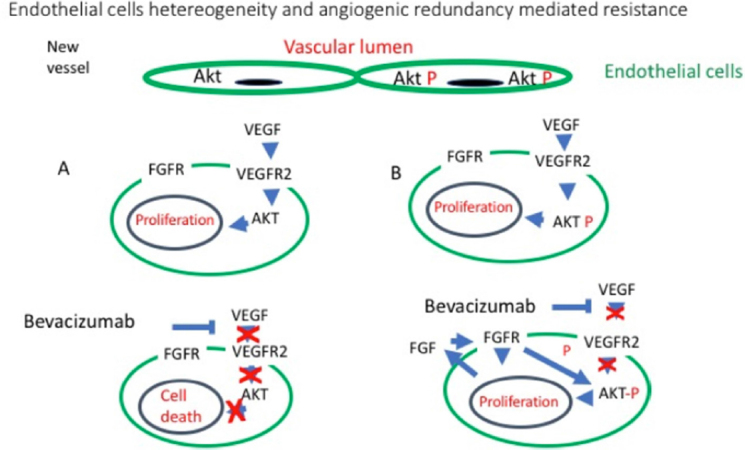
Angiogenic pathways redundancy and endothelial heterogeneity. Intra-tumour endothelial cells, despite being normal, non-neoplastic cells, can be still heterogeneous as far as their biology is concern. Guerrouahen *et al*.^[74]^ have demonstrated that endothelial cells from vessels in ovarian carcinoma can considerably differ as far as phosphorylation of AKT is concerned. A:They describe that, following blockage of *VEGF*, the endothelium with low levels of AKT phosphorylation undergo apoptosis; B: However, in the endothelial cells with high level of phosphor AKT, following the block of *VEGF* stimulation, the AKT pathway remains active inducing transcription of FGF, an angiogenic factor, which on one side promotes endothelium proliferation and on the other further phosphorylates AKT producing a positive loop. (Based on ^[74]^). FGFR: fibroblast growth factor receptor

One example that demonstrates both the effect of angiogenesis redundancy and vascular heterogeneity is the resistance to Bevacizumab observed in ovarian cancer. In human tissue samples of this malignancy, heterogeneity of AKT phosphorylation in the intra tumour endothelial cells has been reported by Guerrouahen et al.^[74]^; they show how within one single vessels, a mixture of heterogeneous endothelial cells is present, as exemplified in Figure 5. To investigate the role of endothelial AKT phosphorylation as a mechanism or resistance “*in vitro*” the authors first selected, trough continuous exposure to Bevacizumab, HUVECs (Human umbilical vein endothelial cells) resistant to Bevacizumab. These cells showed resistance to Bevacizumab “*in vitro*” and also had higher levels of AKT phosphorylation. Treatment with the pan-PI3K inhibitor LY294002 blocked AKT phosphorylation but did not harmed the cells, however abolished the resistance to Bevacizumab as its addiction lead to endothelial cell death indicating that AKT phosphorylation was mediating the resistance to anti *VEGF* treatment. These resistant cells have also higher levels of *FGF2* compared to the normal HUVAC, and following incubation with Bevacizumab, a further increase in *FGF2* transcription and translation was observed alongside an increase in levels of FGFR1 and its phosphorylation. The authors than demonstrated that these higher levels of *FGF2* leads to yet more phosphorylation of AKT alongside activating Src and the pro-angiogenic pathway *ERK1/STAT3* [Figure 5]. As selective inhibition of *FGF2* reverse this process, the authors conclude demonstrating that in these cells, Bevacizumab treatment leads to an autocrine loop supporting the viability of the endothelial cell^[74]^. This intra cellular mechanism can be further enhanced by the recruitment, following Bevacizumab treatment, of marrow-derived fibrocyte-like cells which also produce *FGF2*^[75,76]^.

Increased knowledge of the phenotype of intratumor endothelial cells is now allowing to plan new approaches to overcome the heterogeneity-linked problems met so far^[73]^. One way is to exploit the expression of markers which are more diffusely expressed but are not, by themselves, targets of treatments. By developing petide-ligand motives, and conjugating these peptides to drugs or drug-containing liposome, a broader range of endothelial cells can be targeted^[77,78,73]^. A second approach is to deliver with liposome siRNA which can “switch off” the transcription of factors inducing resistance^[73]^. A third approach is to target the cytoskeleton and the cell-cell junctions of the endothelial cells as these are all structures fairly uniform in endothelial cells^[79]^. Some phase III trials with anti-endothelial compounds have been done but the results have not been encouraging indicating that this could eventually be a rather difficult problem to solve^[80]^.

## Conclusions

The main conclusion that most of these studies share is that resistance to anti angiogenic treatment is very frequent and mediated by several different mechanisms. Therefore if therapies targeting new vessels are going to stay, it will be as part of combined treatments. An emerging new way to use these drugs is in a dual approach, targeting both angiogenic and non-angiogenic growth patterns. Some possible ways to achieve this are emerging. For example, *CD276*, a highly conserved cell-surface protein, is found broadly overexpressed by multiple malignancies on both cancer cells and blood vessels^[44]^. Notably, *CD276* was expressed on both newly formed and pre-existing vessels present inside tumours but not in normal vessels outside the neoplastic mass and not during physiological angiogenesis (e.g., regenerating liver tissue)^[44]^. In a mouse model, an antibody-drug conjugate pyrrobenzodiazepine-conjugated *CD276*, eradicated both large established tumours and metastases and improved long-term overall survival potentially as a result of targeting both angiogenic and non-angiogenic tumour blood vessels^[44]^. However, as these observations are based on animal studies, work is necessary to confirm whether this would be an effective therapy for angiogenic and non-angiogenic human cancers. Progresses on the study of the biology of non-angiogenic tumours and how they co-opt vessels is therefore essential to develop new approaches to cancer treatment but they are likely to be effective only as a part of multi drugs therapeutic protocols.

## Declarations

### Copyright

© The Author(s) 2019.
